# Divergent Pathogenesis and Transmission of Highly Pathogenic Avian Influenza A(H5N1) in Swine

**DOI:** 10.3201/eid3004.231141

**Published:** 2024-04

**Authors:** Bailey Arruda, Amy L. Vincent Baker, Alexandra Buckley, Tavis K. Anderson, Mia Torchetti, Nichole Hines Bergeson, Mary Lea Killian, Kristina Lantz

**Affiliations:** Agriculture Research Service, Ames, Iowa, USA (B. Arruda, A.L. Vincent Baker, A. Buckley, T.K. Andersen);; Animal and Plant Health Inspection Service, Ames (M. Torchetti, N. Hines Bergeson, M.L. Killian, K. Lantz)

**Keywords:** influenza A virus, influenza, viruses, H5N1 subtype, swine, porcine, avian influenza, poultry, mammals, respiratory infections, zoonoses, United States

## Abstract

Highly pathogenic avian influenza (HPAI) viruses have potential to cross species barriers and cause pandemics. Since 2022, HPAI A(H5N1) belonging to the goose/Guangdong 2.3.4.4b hemagglutinin phylogenetic clade have infected poultry, wild birds, and mammals across North America. Continued circulation in birds and infection of multiple mammalian species with strains possessing adaptation mutations increase the risk for infection and subsequent reassortment with influenza A viruses endemic in swine. We assessed the susceptibility of swine to avian and mammalian HPAI H5N1 clade 2.3.4.4b strains using a pathogenesis and transmission model. All strains replicated in the lung of pigs and caused lesions consistent with influenza A infection. However, viral replication in the nasal cavity and transmission was only observed with mammalian isolates. Mammalian adaptation and reassortment may increase the risk for incursion and transmission of HPAI viruses in feral, backyard, or commercial swine.

Influenza A viruses (IAV) of avian and swine origin have caused 5 pandemics in the previous 2 centuries (*1*). Aquatic avian populations, the primary reservoir for IAV, harbor numerous virus subtypes (H1–16), to which mammals have minimal preexisting immunity (*2*). Among those subtypes, H5 avian influenza virus infections have been documented in domestic poultry, humans, marine mammals, and swine, among others (*3*–*6*).

Over the past decade, H5NX highly pathogenic avian influenza (HPAI) viruses belonging to the goose/Guangdong (Gs/GD) 2.3.4.4 hemagglutinin (HA) phylogenetic clade have caused infections in wild birds and poultry, resulting in major mortality events and spread to >84 countries, and were recognized as a panzootic (*7*,*8*). In addition, evidence exists of enzootic HPAI virus maintenance in Europe, further signifying a paradigm shift in the biology of HPAI (*9*). Since February 2022, HPAI H5N1 clade 2.3.4.4b virus originating from a trans-Atlantic incursion has caused outbreaks across North America, resulting in >77 million poultry deaths, extensive deaths in wild bird species, and unprecedented disease in wild mammals (*4*–*6*,*10*)

The transcontinental circulation of clade 2.3.4.4b viruses within bird populations continues to enable reassortment with low pathogenicity avian influenza (LPAI) viruses and resulted in the emergence of numerous genotypes of potentially different phenotypes (*11*,*12*). Furthermore, interspecies transmission between avian species and peridomestic mammals has resulted in viruses with mammalian adaptation markers that pose a public health risk should they gain efficient transmission among mammals. A subset of HPAI H5NX clade 2.3.4.4 viruses bound both α2,6-linked (human) and α2,3-linked (avian) sialic acid receptors (*13*–*16*). Furthermore, nearly half of mammal isolates of HPAI H5N1 clade 2.3.4.4b acquired mammalian adaptation markers (E627K, D701N, or T271A substitutions) in the polymerase basic (PB) 2 protein (*1*,*17*). In addition, the HPAI H5N1 viruses collected during an outbreak in farmed mink contained mutations in the neuraminidase protein that caused disruption of the second sialic acid binding site, a feature typical of human-adapted IAV (*17*). During January 2022–April 17, 2023, a total of 8 reported human cases of H5N1 influenza from clade 2.3.4.4b occurred, many severe or fatal (*4*,*18*). Those characteristics of the current clade 2.3.4.4b H5 HPAI elevate the potential for human infection and adaptation.

A longstanding dogma of IAV biology identified swine as a mixing vessel and vital to the emergence of human pandemic IAV by supporting reassortment that could lead to antigenic shift (*1*). However, at a receptor level, swine might be no more susceptible to infection by avian IAVs than humans (*1*). Mammalian adaption of HPAI is a multigenic trait, and the genetic changes necessary for H5N1 strains to adapt to swine and acquire efficient and sustained transmissibility are poorly understood. However, swine-adapted IAV have a propensity for evolution through polymerase errors and reassortment, followed by spread of mutated or reassorted strains through contact among densely housed commercial pigs and pig transport. If an avian IAV strain, such as H5Nx 2.3.4.4b, successfully infected domestic swine, pig-to-pig transmission, reassortment with endemic swine IAV, or acquisition of adaptive mutations that might enable an avian-to-mammalian switch could potentially occur (*1*). Continued circulation in the wild bird population and peridomestic wild mammal infections elevate the risk for exposure of swine because of the current outbreak’s wide distribution in states with large pig populations. To address concerns over susceptibility of swine to HPAI H5N1 clade 2.3.4.4b virus detected in the United States and to elucidate potential molecular mutations associated with H5N1 replication and transmission in swine, we conducted a study with 4 strains representing 3 different genotypes in a pig pathogenesis and transmission model. This information is key to building awareness and detection capabilities in the swine sector, as well as to informing risk assessments and early warning systems to safeguard human health.

## Materials and Methods

### Viruses

**Figure 1:**
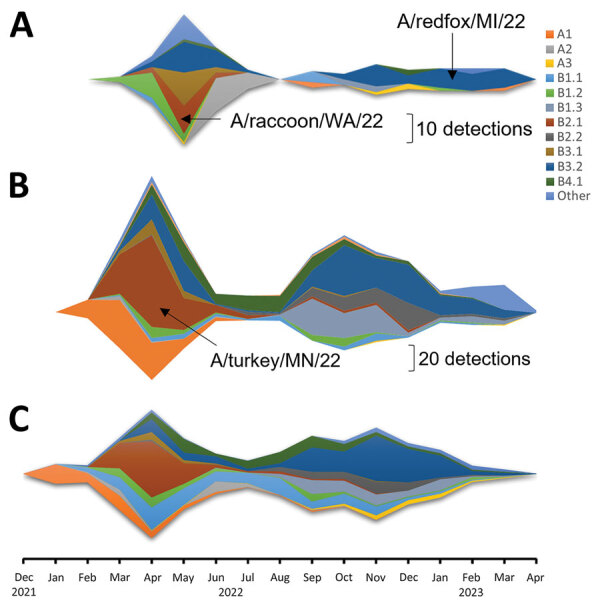
Detection of Eurasian lineage goose/Guangdong H5 clade 2.3.4.4b virus and identification of virus isolates used in study of divergent pathogenesis and transmission of highly pathogenic avian influenza A(H5N1) in swine, by genotype. A) Wild mammal; B) poultry; C) wild birds.

We evaluated the pathogenicity and transmission in crossbred, 4-week-old pigs of 4 strains of the 2022 spring HPAI H5N1 clade 2.3.4.4b outbreak: A/turkey/Minnesota/22–010654-001/2022 (A/turkey/MN/22), A/bald eagle/Florida/W22-134-OP/2022 (A/bald eagle/FL/22), A/raccoon/Washington/22-018406-002/2022 H5N1 (A/raccoon/WA/22) and A/redfox/Michigan/22-018712-001/2022 (A/redfox/MI/22). Those 4 strains resulted from Eurasian avian HPAI H5N1 clade 2.3.4.4b reassortment with North American LPAI lineage internal genes and represented 3 different reassortment patterns frequently detected among H5N1 strains during 2022 (Table 1; Figure 1) (Appendix 1). Both the A/raccoon/WA/22 and the A/redfox/MI/22 strains contained the PB2 E627K mammalian adaptation mutation.

**Table 1:** Virus strain accession number, genotype, constellation, and reassorted genes in study of divergent pathogenesis and transmission of highly pathogenic avian influenza A(H5N1) in swine*

Strain	GISAID accession no.†	Genotype	Constellation (PB2|PB1|PA|HA|NP|NA|M|NS)	Reassorted genes
A/turkey/Minnesota/22-010654-001/2022	16555202	B2.1	am1.2|ea1|ea1|ea1|am1.1|ea1|ea1|ea1	PB2, NP
A/bald eagle/Florida/W22-134-OP/2022	15063846	B1.1	am1.1|am1.1|ea1|ea1|am1.2|ea1|ea1|ea1	PB2, PB1, NP
A/raccoon/Washington/22-018406-002/2022	15078252	B2.1	am1.2|ea1|ea1|ea1|am1.1|ea1|ea1|ea1	PB2, NP
A/red fox/Michigan/22-018712-001/2022	15078253	B3.2	am2.1|am1.2|ea1|ea1|am1.4.1|ea1|ea1|am1.1	PB2, PB1, NP, NS

*am, American; ea, Eurasian; PB2, polymerase basic 2; PB1, polymerase basic 1; PA, polymerase acidic; HA, hemagglutinin; NP, nucleoprotein; NA, neuraminidase; M, matrix protein 1; NS, non-structural protein 1. †GISAID, https://www.gisaid.org.

### Virus Propagation and Titration

We conducted the study in compliance with the Animal Care and Use Committee of the US Department of Agriculture—Agricultural Research Service National Animal Disease Center under Biosafety Level 3 guidelines, including enhancements required by the Federal Select Agent Program. We passaged virus stocks in 10-day-old embryonating chicken eggs, harvested the allantoic fluid from infected eggs, divided it into aliquots, and stored it at –70°C until use. We determined viral titers by using MDCK cells according to standard methods (*19*).

### Swine Pathogenesis and Transmission Study

We blocked 88 pigs by litter and randomly allocated them into a negative control group or a group of 20 (1 group per virus strain). We inoculated 15 pigs per virus strain intranasally with 1 ml (0.5 ml per nostril) of ≈10^5^ 50% tissue culture infective dose/mL using a Nasal Intranasal Mucosal Atomization Device (Teleflex, https://www.teleflex.com). We comingled 5 naive contact pigs with each of the virus-inoculated groups at 2 days postinoculation (dpi). We collected nasal swabs from inoculated pigs and contact pigs on 0, 1, 3, 5, and 7 dpi or days postcontact (dpc). We necropsied 5 inoculated pigs per group at 3, 5, and 14 or 17 dpi and the 5 contact pigs at 12 or 15 dpc. We collected bronchoalveolar lavage fluid (BALF) at necropsy on 3 and 5 dpi. We also obtained serum samples at necropsy (Appendix 1). We necropsied 8 negative control pigs from the same source herd as inoculated and contact pigs on ≈5 DPI to evaluate health status and background respiratory tract lesions.

### Viral RNA Detection and Serology

We extracted viral RNA from nasal swab and BALF samples by using the MagMax Viral RNA isolation kit (ThermoFisher Scientific, https://www.thermofisher.com) and subjected it to real-time reverse transcription assays targeting multiple genes of IAV and a H5 2.3.4.4-specific HA gene (*20*). Cycle threshold (Ct) value for IAV quantitative reverse transcription PCR (qRT-PCR) interpretation was according to manufacturer’s suggestion: Ct value of <38 indicated the sample was positive, Ct value of 38–40 indicated the sample was suspect; if undetected, the sample was negative. We determined seroconversion by using an IAV nucleoprotein (NP)–blocking ELISA (IDEXX, https://www.idexx.com).

### Positive Sample Metagenomic Sequencing and Analyses

We amplified IAV RNA from samples as described (*10*). For each of the samples, we conducted variant calling by trimming raw FASTQ files using Trimmomatic (http://www.usadellab.org/cms/?page=trimmomatic) with a sliding window size of 5 bp and a minimum Q-score of 30, discarding reads that were trimmed to a length <100 bases. We aligned reads to reference sequences using bowtie2 version 2.3.2 (https://sourceforge.net/projects/bowtie-bio/files/bowtie2/2.3.2) and removed duplicate reads were removed using Picard (https://broadinstitute.github.io/picard). We converted the BAM files to mpileup using samtools (https://www.htslib.org) and identified within-host variants using VarScan (https://varscan.sourceforge.net). For a variant to be reported, we required the sequencing depth to be 100×, PHRED quality scores to be 30, and detection frequency to be >1%. We compiled all reported variant calls and raw FASTQ files (https://github.com/flu-crew/datasets). We used the Sequence Feature Variant Types tool from the Influenza Research Database to download all currently available annotations for H5 HA, N1 neuraminidase, and the remaining internal genes (*21*). For each genome, we computed nucleotide diversity using the synonymous (π*_S_*) and nonsynonymous (π*_N_*) diversity calculations in SNPGenie (https://github.com/chasewnelson/SNPGenie) with a minimum allele frequency cutoff set to 1% (*22*) (Appendix 1).

### Macroscopic and Microscopic Lesion Score

At necropsy, we recorded the percentage of affected surface area per lung lobe and used that to calculate a weighted macroscopic lung lesion score (*23*). We fixed tissue samples from the trachea and right middle or affected lung lobe in 10% buffered formalin for histologic examination and transferred to 70% ethanol after 48 hours. A veterinary pathologist blinded to treatment evaluated microscopic lesions of the lung and trachea (Appendix 1).

### Immunohistochemistry

We conducted immunohistochemistry (IHC) staining manually on 5-µm–thick sections using a rabbit polyclonal anti-influenza A NP (GeneTex, https://www.genetex.com) primary antibody. We blocked slide runs by group to account for potential differences between runs and scored as previously described (Appendix 1) (*23*).

### Statistical analysis

We performed all statistical analyses using GraphPad Prism 8.1.2 software (https://www.graphpad.com). We used a Kruskal-Wallis test with Dunn correction for multiple comparisons to compare the weighted macroscopic lung lesion score, microscopic pneumonia score, microscopic tracheitis score, lung IHC score of conducting airways, lung IHC score of nonconducting airways, cumulative lung IHC scores, and tracheal IHC score of virus inoculated groups by dpi. We compared the BALF qRT-PCR Ct values of positive samples using an ordinary 1-way ANOVA with a Šídák multiple comparisons test. We considered an adjusted p value of <0.05 significant in each analysis.

## Results

### Isolate Replication

**Figure 2:**
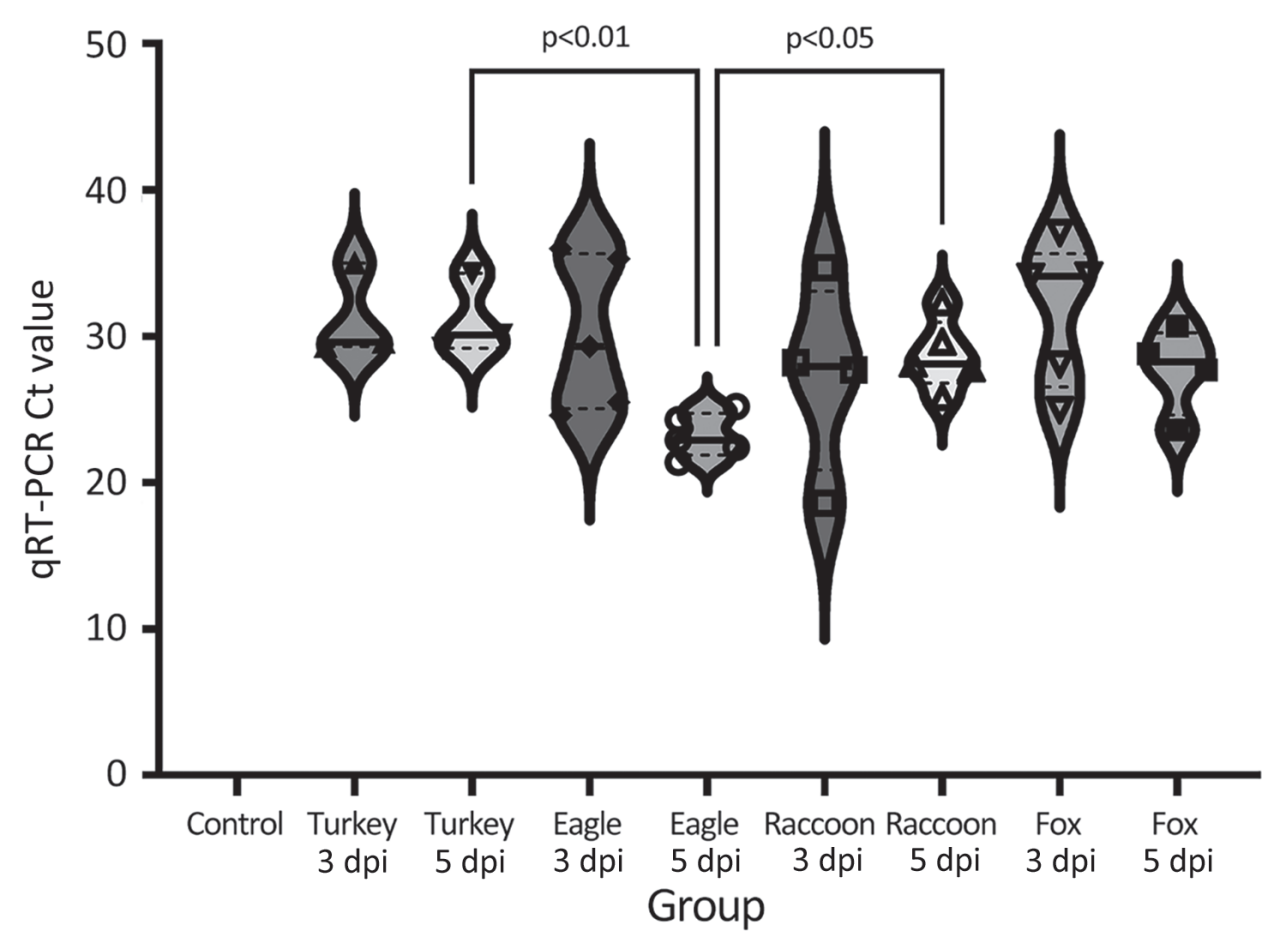
Violin plots showing Ct values for bronchoalveolar lavage fluid from pigs, by virus strain and dpi, in study of divergent pathogenesis and transmission of highly pathogenic avian influenza A(H5N1) in swine. Values were determined by quantitative reverse transcription PCR (ThermoFisher Scientific, https://www.thermofisher.com). All strains replicated in the lung of pigs. No statistical difference was detected between strains by dpi. Solid lines within plots indicate medians, dashed lines indicate quartiles, and symbols indicate individual pig values. dpi, days postinoculation; Eagle 3 dpi, A/bald eagle/FL/22 necropsied on 3 dpi; Eagle 5 dpi, A/bald eagle/FL/22 necropsied at 5 dpi; Fox 3 dpi, A/redfox/MI/22 necropsied at 3 dpi; Fox 5 dpi, A/redfox/MI/22 necropsied at 5 dpi; Racoon 3 dpi, A/raccoon/WA/22 necropsied at 3 dpi; Racoon 5 dpi, A/raccoon/WA/22 necropsied at 5 dpi; Turkey 3 dpi, A/turkey/MN/22 necropsied at 3 dpi; Turkey 5 dpi, A/turkey/MN/22 necropsied at 5 dpi.

All isolates replicated in the lungs of most inoculated pigs, although no overt clinical signs were observed (Figure 2; Appendix 1 Table 1). We detected viral RNA in BALF from 3 of the 5 inoculated pigs necropsied at both 3 and 5 dpi in the A/turkey/MN/22 group, from all inoculated pigs necropsied at both 3 and 5 dpi in the A/bald eagle/FL/22 group, 4 of the 5 inoculated pigs necropsied at both 3 and 5 dpi in the A/raccoon/WA/22 group, and all inoculated pigs necropsied at 3 dpi and 4 inoculated pigs at 5 dpi in the A/redfox/MI/22 group. The lowest group mean Ct value (23.22) was observed in the A/bald eagle/FL/22 group at 5 dpi, followed by the A/raccoon/WA/22 group at 3 dpi (Ct value 26.05) and A/redfox/MI/22 group at 5 dpi (Ct value 26.14). The lowest individual Ct value (18.16) was seen in the A/raccoon/WA/22 group at 3 dpi. We found a significant difference in mean Ct values between the A/bald eagle/FL/22 and A/turkey/MN/22 groups and the A/bald eagle/FL/22 and A/raccoon/WA/22 groups at 5 dpi (p<0.05) (Figure 2). We did not detect viral RNA in BALF samples from control pigs.

### Macroscopic and Microscopic Lesions 

**Figure 3:**
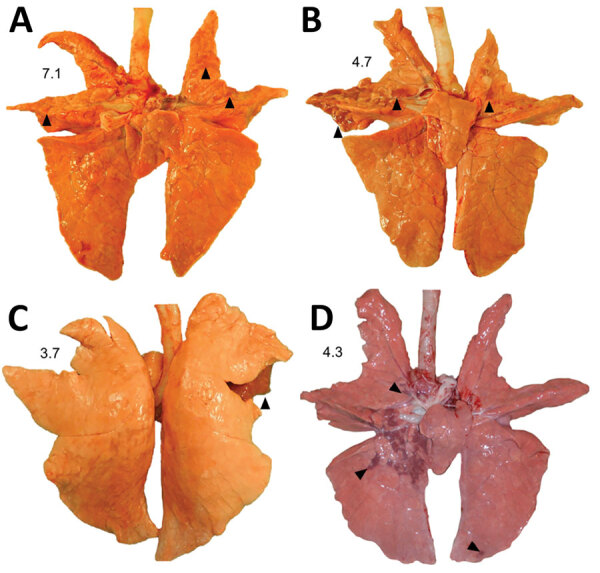
Macroscopic lung lesions and respective weighted macroscopic lung score of swine infected with highly pathogenic avian influenza A(H5N1) virus belonging to the goose/Guangdong 2.3.4.4b hemagglutinin phylogenetic clade. A) Multifocal pulmonary consolidation (arrowheads) in pig 777 infected with A/turkey/MN/22, necropsied at 3 days postinoculation (dpi). B) Multifocal pulmonary consolidation (arrowheads) in pig 796 infected with A/bald eagle/FL/22 necropsied on 5 dpi. C) Locally extensive pulmonary consolidation (arrowheads) in pig 58 infected with A/raccoon/WA/22 necropsied on 3 dpi. D) Multifocal pulmonary consolidation (arrowheads) of pig 78 infected with A/redfox/MI/22 necropsied on 3 dpi.

**Figure 4:**
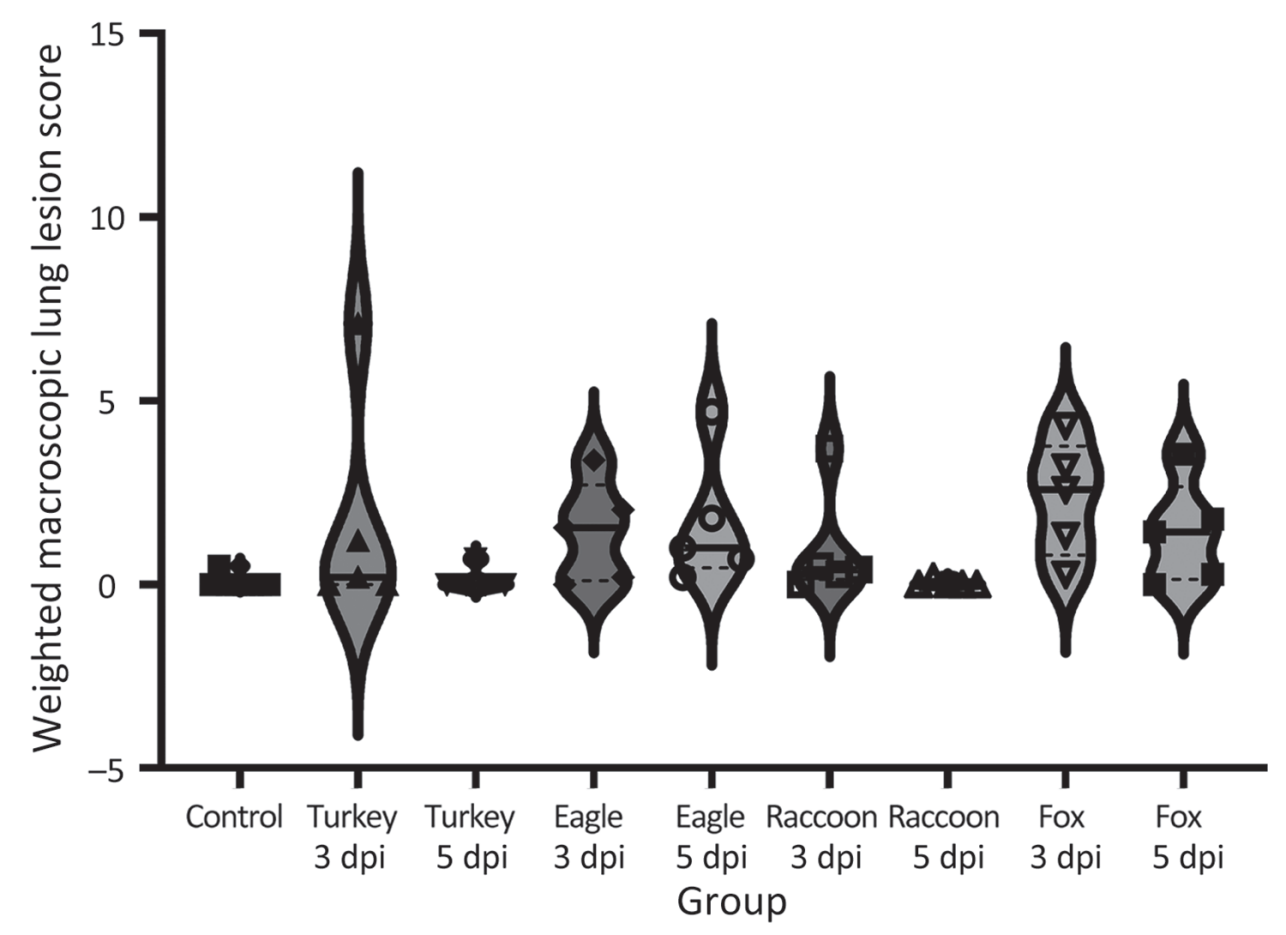
Violin plots of weighted macroscopic lung scores, by virus strain and dpi, of swine infected with highly pathogenic avian influenza A(H5N1) virus belonging to the goose/Guangdong 2.3.4.4b hemagglutinin phylogenetic clade. All strains caused macroscopic lesions consistent with influenza A virus infection in >1 pig. Solid lines within plots indicate medians, dashed lines indicate quartiles, and symbols indicate individual pig values. No statistically significant differences were detected between strains by dpiI. dpi, days postinoculation; eagle 3 dpi, A/bald eagle/FL/22 necropsied at 3 dpi; eagle 5 dpi, A/bald eagle/FL/22 necropsied at 5 dpi; fox 3 dpi, A/redfox/MI/22 necropsied at 3 dpi; fox 5 dpi, A/redfox/MI/22 necropsied at 5 dpi; raccoon 3 dpi, A/raccoon/WA/22 necropsied at 3 dpi; raccoon 5 dpi, A/raccoon/WA/22 necropsied at 5 dpi; turkey 3 dpi, A/turkey/MN/22 necropsied at 3 dpi; turkey 5 dpi, A/turkey/MN/22 necropsied at 5 dpi.

Macroscopic lung lesions consistent with IAV infection developed in pigs in each of the virus-inoculated groups. Macroscopic lesions consisted of multifocal-to-locally extensive predominately cranioventral red-to-purple pulmonary consolidation (Figure 3, panels A–D). Averaging the weighted macroscopic lung lesion score at 3 and 5 dpi by group showed that the A/bald eagle/FL/22 and A/redfox/MI/22 strains caused the highest lesion scores (Figure 4). A significant difference was found between groups necropsied at 5 dpi (p<0.05); however, no significant difference was found in the ad hoc comparisons with only the weighted macroscopic lung lesion score of the A/bald eagle/FL/22 group compared, and the A/turkey/MN/22 at 5 dpi neared significance (p = 0.055).

**Figure 5:**
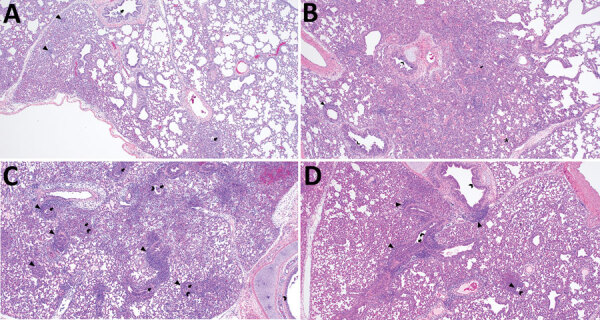
Microscopic lung lesions of swine infected with highly pathogenic avian influenza A(H5N1) belonging to the goose/Guangdong 2.3.4.4b hemagglutinin phylogenetic clade. A) Perivascular mononuclear inflammatory infiltrate (arrowheads) and suppurative bronchiolitis (arrows) in the lung of pig 777 infected with A/turkey/MN/22 necropsied at 3 days postinoculation (dpi). B) Peribronchiolar mononuclear inflammatory infiltrate (arrowhead), suppurative bronchiolitis (arrow), necrotizing bronchiolitis and bronchitis (chevrons), and alveolar luminal accumulation of cellular debris (asterisk) in the lung of pig 796 infected with A/bald eagle/FL/22 necropsied at 5 dpi. C) Peribronchiolar mononuclear inflammatory infiltrate (arrowheads), suppurative bronchiolitis (arrows), and necrotizing bronchiolitis and bronchitis (chevrons) in the lung of pig 58 infected with A/raccoon/WA/22 necropsied at 3 dpi. D) Peribronchiolar and peribronchial mononuclear inflammatory infiltrate (arrowheads), suppurative bronchiolitis (arrow), and necrotizing bronchiolitis and bronchitis (chevrons) in the lung of pig 78 infected with A/redfox/MI/22 necropsied at 3 dpi. Hematoxylin & eosin stain; original magnification ×40.

**Figure 6:**
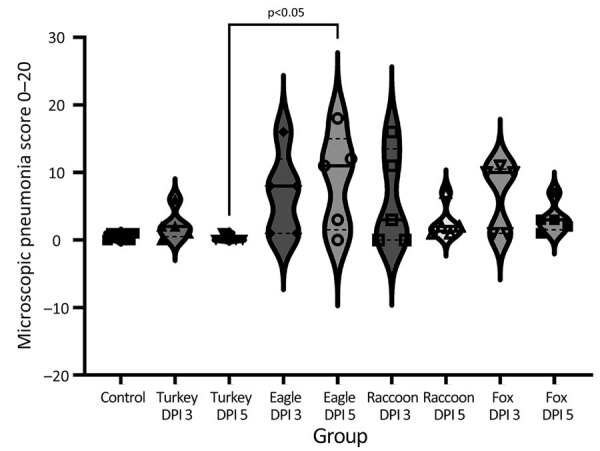
Violin plots of microscopic pneumonia scores, by virus strain and dpi, of swine infected with highly pathogenic avian influenza A(H5N1) virus belonging to the goose/Guangdong 2.3.4.4b hemagglutinin phylogenetic clade. Lesion severity varied by group, with the most severe lesions being observed in the A/bald eagle/FL/22 and A/red fox/MI/22 groups. Solid lines within plots indicate medians, dashed lines indicate quartiles, and symbols indicate individual pig values. dpi, days postinoculation; eagle 3 dpi, A/bald eagle/FL/22 necropsied at 3 dpi; eagle 5 dpi, A/bald eagle/FL/22 necropsied at 5 dpi; fox 3 dpi, A/redfox/MI/22 necropsied at 3 dpi; fox 5 dpi, A/redfox/MI/22 necropsied at 5 dpi; raccoon 3 dpi, A/raccoon/WA/22 necropsied at 3 dpi; raccoon 5 dpi, A/raccoon/WA/22 necropsied at 5 dpi; turkey 3 dpi, A/turkey/MN/22 necropsied at 3 dpi; turkey 5 dpi, A/turkey/MN/22 necropsied at 5 dpi.

Microscopic lung lesions consistent with IAV infection developed in inoculated pigs in each of the virus-inoculated groups; however, the number of pigs with consistent lesions and the severity of lesions varied by group. A/turkey/MN/22 caused little to no microscopic lesions; lesions consistent with IAV infection developed in only 1 of 10 inoculated pigs (Figure 5, panel A). In contrast, lung lesions consistent with IAV infection developed in most pigs in the A/bald eagle/FL/22 group (7 of 10) (Figure 5, panel B). In both the A/raccoon/WA/22 and A/redfox/MI/22 groups, lesions consistent with IAV infection developed in 4 of 10 inoculated pigs (Figure 5, panels C, D). A significant difference was found between the microscopic pneumonia score when comparing the A/turkey/MN/22 DPI 5 group and the A/bald eagle/FL/22 DPI 5 group (p<0.05) (Figure 6). Microscopic tracheitis scores were not statistically different between the virus inoculated groups (Appendix 1 Figure 1).

### Alveolitis and Antigen Labeling 

**Figure 7:**
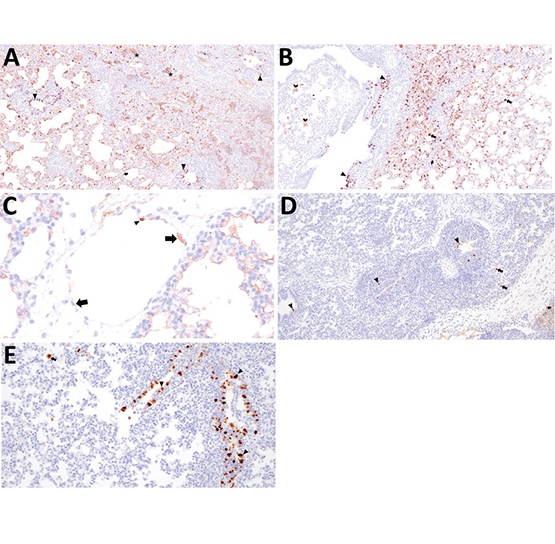
Immunohistochemical detection of influenza A virus nucleoprotein antigen in swine infected with H5N1 highly pathogenic avian influenza belonging to the goose/Guangdong 2.3.4.4b hemagglutinin phylogenetic clade. A) Extensive labeling of pneumocytes lining alveolar septa (arrows) and respiratory epithelium lining bronchioles (arrowheads) in the lung of pig 794 infected with A/bald eagle/FL/22 necropsied at 3 days postinoculation (dpi). Hematoxylin & eosin stain; original magnification ×40. B) Extensive labeling of pneumocytes lining alveolar septa (arrow), respiratory epithelium lining a bronchus (arrowheads), cell membrane of alveolar macrophages (chevron), and within the cytoplasm and nucleus of alveolar macrophages consistent with viral replication (notched arrow) in the lung of pig 798 infected with A/bald eagle/FL/22 necropsied on 5 dpi. Hematoxylin & eosin stain; original magnification ×40. C) Labeling in the cytoplasm (arrows) and nucleus (arrowhead) of endothelial cells in the lung of pig 791 infected with A/bald eagle/FL/22 necropsied on 3 dpi. Hematoxylin & eosin stain; original magnification ×200. D) Labeling of respiratory epithelium lining a bronchus (arrowheads), within the cytoplasm and nucleus of alveolar macrophages consistent with viral replication (notched arrow), rarely pneumocytes (arrow), in the lung of pig 58 infected with A/raccoon/WA/22 necropsied on 3 dpi. Hematoxylin & eosin stain; original magnification ×40. E) Abundant labeling of respiratory epithelium lining a bronchiole (arrowheads) and within the cytoplasm and nucleus of alveolar macrophages consistent with viral replication (notched arrow) in the lung of pig 78 infected with A/redfox/MI/22 necropsied on 3 dpi. Hematoxylin & eosin stain; original magnification ×100.

Divergent pathogenesis between HPAI strains was further evidenced by the extent of alveolitis and differential distribution and abundance of NP antigen by IHC. We did not detect HPAI NP antigen in any lung section from the A/turkey/MN/22 group but did detect HPAI NP antigen in the trachea of 2 pigs from this group. In contrast, we detected HPAI NP antigen in the trachea (8 of 10) and lung (7 of 10) in pigs in the A/bald eagle/FL/22 group. We also detected antigen in the respiratory epithelium of conducting airways, macrophages, pneumocytes, alveolar luminal debris, and, rarely, endothelial cells of pigs inoculated with A/bald eagle/FL/22 (Figure 7, panels A–C). In addition, the degree of alveolitis characterized by extensive widening of alveolar septa because of a mononuclear inflammatory infiltrate and luminal accumulation of edema and cellular debris, a change not typical of swine-adapted IAV, was most prominent in the A/bald eagle/FL/22 group (Figure 7, panels A, B).

**Figure 8:**
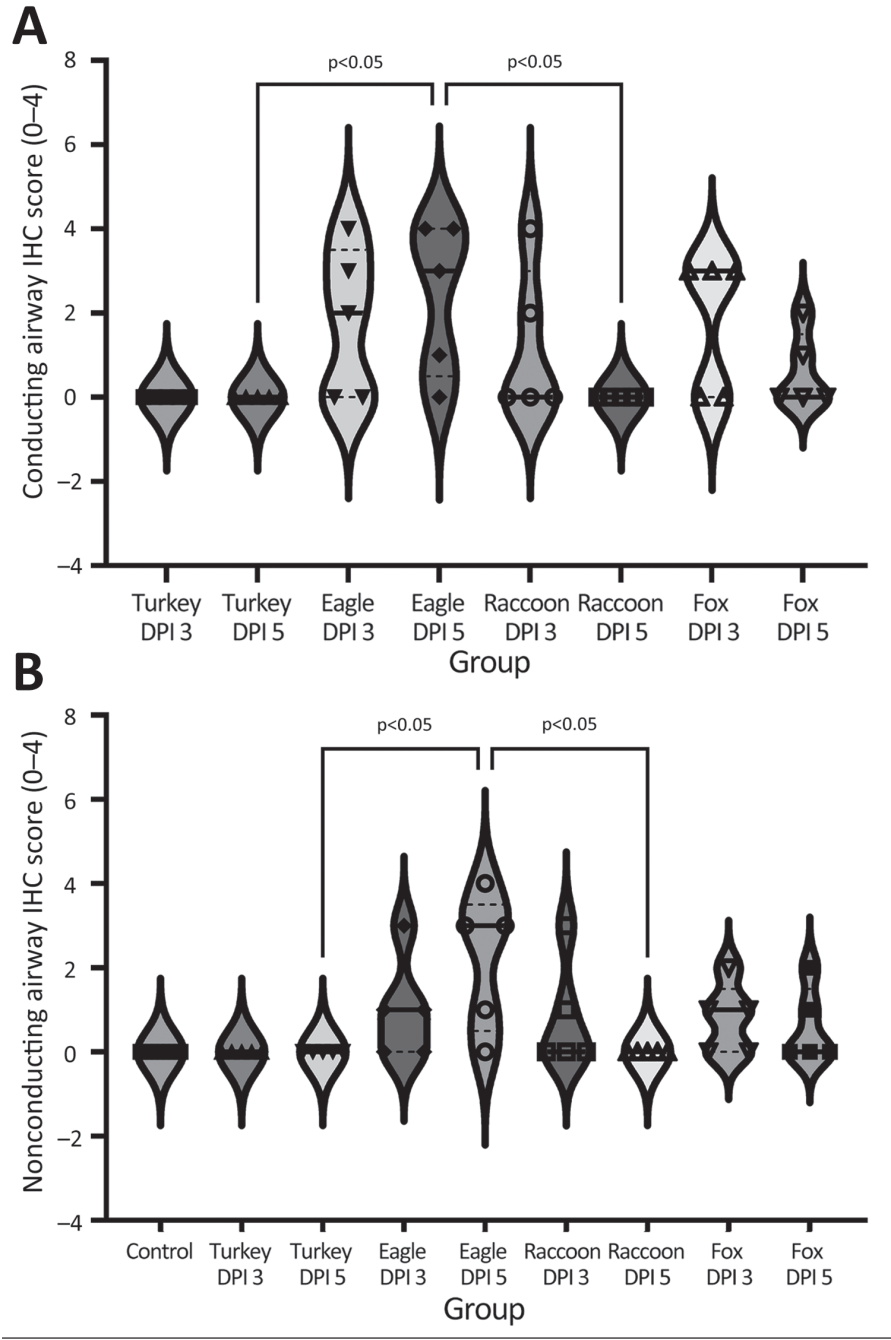
Violin plots of airway conducting IHC scores, by virus strain and dpi, of swine infected with highly pathogenic avian influenza A(H5N1) virus belonging to the goose/Guangdong 2.3.4.4b hemagglutinin phylogenetic clade. A) Lung conducting airway immunohistochemical scores. Influenza A virus (IAV) nucleoprotein (NP) antigen detection varied by group with the most extensive labeling (the number of positive pigs) being observed in the A/bald eagle/FL/22 and A/red fox/MI/22 groups. B) Lung nonconducting airway immunohistochemical scores. IAV NP antigen detection varied by group with the most extensive labeling being observed in the A/bald eagle/FL/22. Solid lines within plots indicate medians, dashed lines indicate quartiles, and symbols indicate individual pig values. dpi, days postinoculation; eagle 3 dpi, A/bald eagle/FL/22 necropsied at 3 dpi; eagle 5 dpi, A/bald eagle/FL/22 necropsied at 5 dpi; fox 3 dpi, A/redfox/MI/22 necropsied at 3 dpi; fox 5 dpi, A/redfox/MI/22 necropsied at 5 dpi; IHC, immunohistochemistry; racoon 3 dpi, A/raccoon/WA/22 necropsied at 3 dpi; racoon 5 dpi, A/raccoon/WA/22 necropsied at 5 dpi; turkey 3 dpi, A/turkey/MN/22 necropsied at 3 dpi; turkey 5 dpi, A/turkey/MN/22 necropsied at 5 dpi.

We detected HPAI NP antigen in the trachea (6 of 10) or lung (2 of 10) of pigs from the A/raccoon/WA/22 group and trachea (7 of 10) or lung (5 of 10) of pigs in the A/redfox/MI/22 group. However, the distribution of antigen in those 2 groups varied compared to the A/bald eagle/FL/22 group. NP antigen was less commonly observed in macrophages, pneumocytes, and alveolar luminal debris and not observed in endothelial cells (Figure 7, panels D, E). We observed significant differences among the conducting airway (Figure 8, panel A), nonconducting airway (Figure 8, panel B), and cumulative lung IHC scores (Appendix 1 Figure 2) of the A/bald eagle/FL/22 5 dpi group and both the A/turkey/MN/22 group and A/raccoon/WA/22 5 dpi groups (p<0.05). We observed no significant difference for tracheal IHC score between the virus-inoculated groups (Appendix 1 Figure 3). We did not detect NP antigen in any samples from control pigs.

### Mammalian Isolates 

Neither A/turkey/MN/22 nor A/bald eagle/FL/22 replicated to detectable levels in the nasal cavity of inoculated pigs (0 of 15 per strain) or transmitted on the basis of seroconversion or detection of viral RNA in nasal swab samples from direct-contact pigs (0 of 5 per strain) (Table 2). In contrast, we detected A/raccoon/WA/22 in the nasal cavity of inoculated pigs (4 of 15) and transmitted to contacts (2 of 5). Similarly, we detected A/redfox/MI/22 in the nasal cavity of inoculated pigs (5 of 15) and transmitted to a single contact (Table 3). We did not detect viral RNA in any nasal swab samples from control pigs.

**Table 2:** Replication and transmission data in study of divergent pathogenesis and transmission of highly pathogenic avian influenza A(H5N1) in swine*

Strain	Inoculated pigs					Contact pigs	
	NS PCR-positive	BALF PCR-positive, 3 dpi	BALF PCR-positive, 5 dpi	Seroconversion		NS PCR-positive	Seroconversion
A/turkey/MN/22	0/15	3/5	3/5	2/5		0/5	0/5
A/bald eagle/FL/22	0/15	5/5	5/5	3/5		0/5	0/5
A/raccoon/WA/22	4/15	4/5	5/5	5/5		2/5	1/5
A/red fox/MI/22	5/15	5/5	4/5	5/5		0/5	1/5

*Nasal swab samples were taken on 0, 1, 3, 5, and 7 dpi or dpc. BALF was collected at 3 and 5 dpi. Influenza A virus qRT-PCR. Seroconversion results of inoculated pigs are only on surviving pigs at ≈2 weeks dpi or dpc. BALF, bronchoalveolar lavage fluid; dpc, days postcontact; dpi, days postinoculation; NS, nasal swab; qRT-PCR, quantitative reverse transcription PCR.

**Table 3:** Ct values for nasal swab samples tested for influenza A virus by qRT-PCR by pig and days postinoculation for A/raccoon/WA/22 and A/redfox/MI/22 strains

Pig ID	Days postinoculation or postcontact				
	0	1	3	5	7
A/raccoon/WA/22					
56	ND	ND	ND	NA	NA
57	ND	ND	ND	NA	NA
58	ND	ND	ND	NA	NA
59	ND	ND	ND	NA	NA
60	ND	ND	ND	NA	NA
61	ND	33.0	34.7	33.2	NA
62	ND	ND	ND	ND	NA
63	ND	ND	ND	38.8	NA
64	ND	ND	ND	ND	NA
65	ND	ND	ND	32.1	NA
66	ND	ND	ND	ND	ND
67	ND	ND	ND	ND	29.5
68	ND	ND	ND	ND	ND
69	ND	36.5	ND	36.9	35.1
70	ND	ND	ND	ND	ND
71	ND	ND	ND	ND	38.1
72	ND	ND	ND	ND	32.5
73	ND	ND	ND	ND	35.0
74	ND	ND	ND	ND	ND
75	ND	ND	ND	ND	ND
A/redfox/MI/22					
76	ND	ND	ND	NA	NA
77	ND	ND	ND	NA	NA
78	ND	ND	ND	NA	NA
79	ND	ND	ND	NA	NA
80	ND	ND	ND	NA	NA
81	ND	ND	ND	ND	NA
82	ND	ND	ND	ND	NA
83	ND	ND	ND	ND	NA
84	ND	ND	ND	ND	NA
85	ND	ND	34.8	ND	NA
86	ND	ND	28.1	39.2	37.1
87	ND	ND	ND	ND	37.9
88	ND	ND	ND	36.0	ND
89	ND	ND	ND	35.4	ND
90	ND	ND	ND	38.9	ND
91	ND	ND	ND	ND	ND
92	ND	ND	ND	ND	ND
93	ND	ND	ND	ND	ND
94	ND	ND	ND	ND	ND
95	ND	ND	ND	ND	ND

*Ct value of <38.0 indicates a positive sample, 38–40 indicates suspect sample, and ND indicates negative sample. Pigs numbered 71–75 and 91–95 are contact pigs. Ct, cycle threshold; ID, identification; NA, not available (pig previously euthanized as part of study design); ND, not detected; qRT-PCR, quantitative reverse transcription PCR.

We identified within-host variants in PCR-positive samples across the genome for the 4 strains during infection and after transmission that were present in >1% of sequencing reads (Appendix 1 Table 2). Most single-nucleotide variants were present at low frequencies (Appendix 2). Of the polymorphic amino acid sites, 41 nonsynonymous mutations occurred at sites associated with functional changes, including PB2 E627K detected as a minor variant (4.95%) in A/turkey/MN/22 at 5 dpi in a single sample (Appendix 2). We also detected Polymorphisms at low levels in the A/bald eagle/FL/22 and A/turkey/MN/22 HA gene at position 239 (R239H and R239C). We also detected HA mutations associated with receptor binding affinity at low levels in single samples in A/redfox/MI/22 (S110N, P139L, L513S) and A/raccoon/WA/22 (S110N, M404V, E267K).

We calculated synonymous (π*_S_*) and nonsynonymous (π*_N_*) polymorphisms to assess selection; we considered π*_N_/*π*_S_ <*1 suggestive of purifying selection and π*_N_/*π*_S_ >*1 suggestive of positive selection. When we combined the diversity estimates across genes, all strains exhibited π*_N_/*π*_S_ <*1 (A/turkey/MN/22 π*_N_/*π*_S_* = 0.36; A/bald eagle/FL/22 π*_N_/*π*_S_* = 0.40; A/redfox/MI/22 π*_N_/*π*_S_* = 0.50; A/raccoon/WA/22 π*_N_/*π*_S_* = 0.74), suggesting that the within-host populations tended to exhibit weak purifying selection.

## Discussion

We conducted a pathogenesis and transmission study to understand the susceptibility of swine to 3 genotypes of HPAI H5N1 belonging to the goose/Guangdong 2.3.4.4b HA phylogenetic clade detected within the United States. Our data demonstrated that pigs are susceptible to infection. All 4 HPAI isolates that were evaluated replicated in the lungs of pigs. In comparison to an H1N1 swine-adapted virus, the qRT-PCR Ct values in BALF of the 4 HPAI strains were lower (≈3–8 Ct), except for the A/bald eagle/FL/22 (genotype B1.1) 5 dpi group (Appendix 1 Table 3) (*24*). Replication in the nasal cavity and transmission occurred only in the A/raccoon/WA/22 (genotype B2.1) and A/redfox/MI/22 (genotype B3.2) groups containing the mammalian adaptation mutation E627K in the PB2 gene. However, the number of pigs with qRT-PCR–positive nasal swabs was considerably than for the swine-adapted virus; the approximate viral load was lower (≈4–6 Ct), and detection was later (1 dpi vs. 5 or 7 dpi) (Appendix 1 Table 4) (*24*). Pig 61 (relatively early detection) might have infected inoculated cohorts, and transmission to contacts at later points not captured in our study design might have occurred. The finding of replication of HPAI H5NX clade 2.3.4.4x in the lung of pigs in an experimental model has been shown, but demonstrating replication in the nasal cavity and transmission to contact pigs is novel (*25*,*26*). In addition, a recent study deemed pigs to be highly resistant to clade 2.3.4.4b infection, illustrating the need for continued assessment of genetically diverse viruses with variable phenotypes in pigs (*27*).

The effects of HPAI viruses can range from asymptomatic infections to severe disease in mammals (*28*–*31*). Multiple viral proteins contribute to the pathogenicity and transmissibility of HPAI, and a combination of adaptive mutations and reassortment are likely necessary for efficient mammal transmission (*32*). Both mammal isolates evaluated in this study contained the PB2 E627K mutation, were detected in the noses of inoculated pigs, and transmitted to >1 contact pig. The PB2 gene of all human seasonal viruses of the 20th century contain K627, whereas most clade 2.3.4.4b viruses detected in birds in 2022–2023 contain E627, supporting the role of that mutation in mammalian adaptation (*1*,*17*). Although we did not fully evaluate the direct effects of the E627K mutation in swine, the shedding and transmission profile shown for the 2 mammal isolates in this study indicate this adaptive mutation might have increased viral fitness through enhanced polymerase activity to enable transmission in an otherwise less susceptible host.

Whether different internal gene constellations or other genotypic differences between A/bald eagle/FL/22 (North American PB2, PB1, and NP) and A/turkey/MN/22 (North American PB2 and NP; genotype 2.1) are responsible for the pathogenic differences of those isolates in pigs is unknown. The number of North American gene segments in reassorted HPAI H5N1 clade 2.3.4.4b avian isolates and disease severity in mammals was suggested to have a positive correlation in ferret and mice models (*11*). Although we did not observe evidence of shedding or transmission, both avian isolates replicated in the lung, which is concerning because of potential reassortment with endemic swine viruses.

Viral populations were dominated by low-frequency (<5%) variation that appeared to be shaped by purifying selection. We detected a subset of mutations associated with human receptor binding and specificity (HA S110N, P139L, R239C/H, E267K, L513S) and mammalian replication (PB2 E627K). However, the detected mutations remained at low frequency, and those present early were not transmitted, did not persist, and were not consistently detected across animals. Those data are in accordance with previous studies documenting within-host evolution of H5N1 in poultry (*33*–*35*). Consequently, though adaptive mutations might occur during H5N1 infection in pigs, because of the short infection time and presence of purifying selection (π*_N_/*π*_S_ <*1), the evolutionary potential of the strains in this study appears limited, and the functional effects for the documented mutations require additional study.

The HA proteins of HPAI H5N1 2.3.4.4b virus preferentially bind to α2,3-linked sialic acids on the host cell (*11*), which are at low abundance in the porcine upper respiratory system (*32*). The low abundance of α2,3-linked sialic acids on epithelial cells in the pigs’ nasal cavities might explain why HPAI avian isolates did not transmit. The quantity of α2,3-linked sialic acids is relatively higher in the lungs of pigs and humans and localized to pneumocytes and nonciliated bronchiolar cells (*36*–*39*). That distribution is consistent with the extent and distribution of IHC IAV NP labeling in the lung of pigs inoculated with A/bald eagle/FL/22; we noted prominent alveolitis in those pigs, in contrast to those inoculated with either A/raccoon/WA/22 or A/redfox/MI/22.

Interspecies spillovers commonly result in dead-end infections because the virus likely requires multiple transmission events to acquire the necessary adaptive mutations (*40*). The probability of a virus acquiring a complete set of adaptive mutations in a single immunocompetent host with onward transmission is extremely low (*34*). However, continued circulation of HPAI strains that have already adapted within various mammalian species makes that possibility more likely (*1*,*34*). On-farm transmission among pigs in Indonesia of an HPAI H5N1 and identification of a purified clone with the ability to recognize α2,6 sialic acid receptors were reported (*3*). More recently, serologic evidence of infection of domestic pigs with clade 2.3.4.4b was reported (*41*). In addition, because reassortment occurred with the past 4 influenza pandemics, the propensity for reassortment in swine may increase the risk for H5N1 adaptation toward humans, particularly with the maintenance of 2009 pandemic H1N1 human seasonal virus genes in pigs (*42*). Although infrequent, incursion of LPAI into commercial swine herds in North America occurs periodically, yet the sources of incursion often remain unknown (*43*–*45*). Increased viral fitness characterized by transmission of LPAI strains after reassortment with swine-adapted IAV in pigs was demonstrated both in commercial swine herds and experimentally (*43*,*46*).

The genetic attributes that resulted in the continued circulation of the HPAI H5N1 2.3.4.4b lineage are not well understood. Repeated spillover and spillback events resulted in genotypically and phenotypically diverse reassortant viruses, some of which caused neurologic disease in mammals, a manifestation not observed in pigs (*47*). However, detection of NP antigen in endothelial cells of pigs infected with A/bald eagle/FL/22 suggests this strain might spread systemically.

The risk for reassortment of the HPAI H5N1 2.3.4.4b lineage with endemic swine IAV is a consideration on the basis of the susceptibility to this lineage demonstrated in our study, the prevalence of IAV infection and comorbidities in swine herds, and animal husbandry practices (*48*,*49*). However, the risk for incursion is likely lower in confinement operations with industry standard biosecurity than for backyard or feral pigs. Birdproofing feed and facilities, avoiding the use of untreated water, and restricting peridomestic scavenger mammals from premises are measures to increase biosecurity against HPAI H5N1 clade 2.3.4.4b virus incursion into swine herds.

Appendix 1 Additional information about divergent pathogenesis and transmission of highly pathogenic avian influenza A(H5N1) in swine.

Appendix 2 Additional data from study about divergent pathogenesis and transmission of highly pathogenic avian influenza A(H5N1) in swine.
