# Supplementary material for: Divergent Pathogenesis and Transmission of Highly Pathogenic Avian Influenza A(H5N1) in Swine
Source: Emerg Infect Dis. 2024 Apr;30(4):738–51. doi: 10.3201/eid3004.231141 (PMC10977838; doi:10.3201/eid3004.231141)
Supplement: Appendix 1 — Additional information about divergent pathogenesis and transmission of highly pathogenic avian influenza A(H5N1) in swine. [file 23-1141-Techapp-s1.pdf]

Article DOI: https://doi.org/10.3201/eid3004.231141

EID cannot ensure accessibility for supplementary materials supplied by authors. Readers who have difficulty accessing supplementary content should contact the authors for assistance.

Divergent Pathogenesis and Transmission of Highly Pathogenic Avian Influenza A(H5N1) in Swine

Appendix

Additional Materials and Methods

Viruses

All strains retained the H5 and N1 from the 2.3.4.4b lineage. A/turkey/Minnesota/2022 contained North American LPAI PB2 and NP and is classified as a genotype B2.1. A/bald eagle/Florida/2022 contained North American LPAI PB2, PB1, and NP and is classified as a genotype B1.1 (*1*). Neither the A/turkey/Minnesota/2022 nor the A/bald eagle/Florida/2022 strains contained known mammalian adaptation mutations. A/raccoon/WA/22 contained North American LPAI PB2 and NP and is classified as a genotype B2.1. A/redfox/MI/22 contained North American LPAI PB1, PB2, NP, and NS and is classified as a genotype B3.2.

Swine pathogenesis and transmission study

Eighty-eight pigs were obtained from a herd free of influenza A virus (IAV), porcine reproductive and respiratory syndrome virus, and *Mycoplasma hyopneumoniae*. Prior to inoculation, pigs were confirmed seronegative to IAV by a blocking ELISA (IDEXX) and treated with tulathromycin (Zoetis) and ceftiofur crystalline free acid (Zoetis). Pigs were blocked by litter and randomly allocated using the Excel random function into a negative control group or a group of 20 (one group per virus strain), housed in individual containment rooms. Five naive contact pigs were comingled with each of the intranasally virus-inoculated groups at 2 days post-inoculation (DPI). Nasal swabs (Minitip FLOQSwabs) samples were collected on 0, 1, 3, 5, and 7 DPI from inoculated pigs and 1, 3, 5 and 7 days post-contact (DPC) from contact pigs. Five

inoculated pigs per group were necropsied at 3, 5 and 14 or 17 DPI. The five contact pigs were necropsied at 12 or 15 DPC. Bronchoalveolar lavage fluid (BALF) was collected from inoculated pigs at necropsy on 3 and 5 DPI. Sera were obtained from inoculated pigs and contact pigs at necropsy. All pigs were cared for in compliance with the Institutional Animal Care and Use Committee of the National Animal Disease Center.

H5 2.3.4.4-lineage specific RRT-PCR

The real-time RT-PCR targeting IAV H5 2.3.4.4-lineage viruses assay generates a 115 bp amplicon using two forward primers H5+800/T and H5+800/A (5′- GAG AGT AAT GGA AAT TTY ATT GCT CC-3′ and 5′- GAG AGT AAT GGA AAT TTY ATT GCA CC-3′, respectively), reverse primer H5–915 (5′- GTT TGA CAT TTG GTG TTG CA-3′), and the detection probe H5+855 (5′-[FAM]- GGG ACT CAA CAA TYA TGA AAA GTG -[NFQ]-3′). Y (C or T) indicates positions with multiple nucleotides. AgPath-ID One-Step RT-PCR kit was used to prepare a 25 µl reaction mixture using 1 µl of kit-supplied enzyme mix, 12.5 µl of kit-supplied buffer (2X), 5 pmol each of forward primers and detection probe and 10 pmol of reverse primer. The RT step conditions were 10 min at 45°C and 10 min at 95°C followed by two-step PCR cycling for 40 cycles of 94°C for 10 sec, 57°C for 30 sec, and 72°C for 10 sec. Fluorescence data were acquired at the end of the annealing step.

Positive sample metagenomic sequencing and analyses

Briefly, cDNA libraries for MiSeq were generated using the Nextera XT DNA Sample Preparation Kit (Illumina, https://www.illumina.comExternal Link) and sequenced using the 500 cycle MiSeq Reagent Kit v2 (Illumina) according to manufacturer instructions. De novo and directed assembly of genome sequences was performed using IRMA version 0.6.7, followed by visual verification in DNAStar SeqMan version 14 (https://www.dnastar.com) as needed. For each of the samples, variant calling was conducted by trimming raw FASTQ files using Trimmomatic using a sliding window size of 5 bp, a minimum Q-score of 30, and reads that were trimmed to a length shorter than 100 bases were discarded (*2*). These reads were aligned to reference sequences using bowtie2 v2.3.2 (*3*), duplicate reads were removed using Picard. The BAM files were converted to mpileup with samtools (*4*), and within-host variants were identified using VarScan (*5*). For a variant to be reported, we required the sequencing depth to be 100x, PHRED quality scores of 30, and to be detected at a frequency of at least 1% following thresholds that have been applied in prior studies (*6*). Nonsynonymous changes and those that

had been associated with a functional change were annotated with the results provided for each strain: the HA gene was annotated using H5 numbering including the signal peptide.

Microscopic lesion score

Microscopic lesion score was performed as previously described (*7*,*8*). Five parameters were evaluated per lung section: necrotizing bronchitis and bronchiolitis, suppurative bronchitis and bronchiolitis, peribronchiolar and perivascular lymphocytic infiltrates, alveolar septal inflammation and alveolar cellular exudate, edema, and hemorrhage. A composite score was computed with the sum of the five individual scores (0–20) (*7*). Two parameters were evaluated per tracheal section: epithelial degeneration and necrosis and inflammatory infiltrate for a possible composite score of 0–8 (*8*). Microscopic lesions were considered consistent with IAV infection if the microscopic lesion score was greater than 6 (lung) or 5 (trachea) and IAV was detected via PCR in BALF.

Immunohistochemistry

Heat-induced epitope retrieval was performed in an EZ-Retriever® IR System (BioGenex, Fremont, CA) using a citrate buffer (pH 6.0; Abcam) heated to 95°C for 2 cycles of 10 minutes. Slides were allowed to cool for 20 minutes followed by a rinse with deionized water and Dako Wash Buffer (2 × 5 min; Agilent). Slides were then incubated with hydrogen peroxide (3% in PBS; 2 X 8 minutes) to quench endogenous peroxidase activity, washed, and blocked for 60 minutes with 10% goat serum diluted in Dako Wash Buffer. Slides were incubated at room temperature for 60 minutes with primary antibody (1:2000) diluted in antibody dilution buffer (Agilent), followed by a secondary antibody (EnVision+ anti-rabbit Poly-HRP-IgG; Agilent) for 30 minutes. Diaminobenzidine chromogen detection was completed using Dako DAB Plus (5 min; Agilent) and Dako DAB Enhancer (3 min; Agilent). Slides were then rinsed with deionized water, cleared through gradient alcohol and xylene, counterstained using hematoxylin and coverslipped. Sections were examined by a veterinary pathologist (BA) using an Olympus BX43 light microscope. Photomicrographs were taken using an Olympus DP28 camera. Lung (conducting airways and alveolar lumen and septa) and tracheal immunohistochemical (IHC) score was recorded for each section as previously described (*9*).

Results

Mammalian and avian isolate within-host diversity was dominated by low-frequency variation

We identified 276 SNVs in the A/redfox/MI/22 (170 nonsynonymous, 102 synonymous, 4 missense), 203 SNVs in A/raccoon/WA/22 (120 nonsynonymous, 77 synonymous, 6 missense), 139 SNVs in A/bald eagle/FL/22 (87 nonsynonymous, 51 synonymous, 1 missense), and 51 SNVs in A/turkey/MN/22 (29 nonsynonymous, 22 synonymous).

References

1. Youk S, Torchetti MK, Lantz K, Lenoch JB, Killian ML, Leyson C, et al. H5N1 highly pathogenic avian influenza clade 2.3.4.4b in wild and domestic birds: introductions into the United States and reassortments, December 2021–April 2022. Virology. 2023;587:109860. **PMID 37572517**

2. Bolger AM, Lohse M, Usadel B. Trimmomatic: a flexible trimmer for Illumina sequence data. Bioinformatics. 2014;30:2114–20. PubMed https://doi.org/10.1093/bioinformatics/btu170

3. Langmead B, Salzberg SL. Fast gapped-read alignment with Bowtie 2. Nat Methods. 2012;9:357–9. PubMed https://doi.org/10.1038/nmeth.1923

4. Li H, Handsaker B, Wysoker A, Fennell T, Ruan J, Homer N, et al.; 1000 Genome Project Data Processing Subgroup. The Sequence Alignment/Map format and SAMtools. Bioinformatics. 2009;25:2078–9. PubMed https://doi.org/10.1093/bioinformatics/btp352

5. Koboldt DC, Chen K, Wylie T, Larson DE, McLellan MD, Mardis ER, et al. VarScan: variant detection in massively parallel sequencing of individual and pooled samples. Bioinformatics. 2009;25:2283–5. PubMed https://doi.org/10.1093/bioinformatics/btp373

6. Moncla LH, Bedford T, Dussart P, Horm SV, Rith S, Buchy P, et al. Quantifying within-host diversity of H5N1 influenza viruses in humans and poultry in Cambodia. PLoS Pathog. 2020;16:e1008191. PubMed https://doi.org/10.1371/journal.ppat.1008191

7. Morgan SB, Hemmink JD, Porter E, Harley R, Shelton H, Aramouni M, et al. Aerosol delivery of a candidate universal influenza vaccine reduces viral load in pigs challenged with pandemic H1N1 virus. J Immunol. 2016;196:5014–23. PubMed https://doi.org/10.4049/jimmunol.1502632

8. Gauger PC, Loving CL, Khurana S, Lorusso A, Perez DR, Kehrli ME Jr, et al. Live attenuated influenza A virus vaccine protects against A(H1N1)pdm09 heterologous challenge without vaccine associated enhanced respiratory disease. Virology. 2014;471-473:93–104. PubMed https://doi.org/10.1016/j.virol.2014.10.003

9. Gauger PC, Vincent AL, Loving CL, Henningson JN, Lager KM, Janke BH, et al. Kinetics of lung lesion development and pro-inflammatory cytokine response in pigs with vaccine-associated enhanced respiratory disease induced by challenge with pandemic (2009) A/H1N1 influenza virus. Vet Pathol. 2012;49:900–12. PubMed https://doi.org/10.1177/0300985812439724

10. Arruda BL, Kanefsky RA, Hau S, Janzen GM, Anderson TK, Vincent Baker AL. Mucin 4 is a cellular biomarker of necrotizing bronchiolitis in influenza A virus infection. Microbes Infect. 2023;25:105169. PubMed https://doi.org/10.1016/j.micinf.2023.105169

Appendix 1 Table 1. RT-qPCR Ct value and pathologic data by group and pig identification*

Group	Pig ID	BALF IAV PCR Ct	BALF H5 2.3.4.4 PCR Ct	Weighted Macroscopic Lung Lesion Score	Microscopic Pneumonia Score (0–20)	Conducting Airway Lung IHC Score (0–4)	Non-conducting Airway Lung IHC Score (0–4)	Cumulative Lung IHC Score (0–8)	Microscopic Tracheitis Score (0–8)	Trachea IHC Score (0–4)
Negative control	797	Neg	Neg	0.00	1	0	0	0	0	0
Negative control	816	Neg	Neg	0.50	1	0	0	0	0	0
Negative control	817	Neg	Neg	0.00	1	0	0	0	0	0
Negative control	51	Neg	Neg	0.00	1	0	0	0	1	0
Negative control	52	Neg	Neg	0.00	0	0	0	0	1	0
Negative control	53	Neg	Neg	0.00	0	0	0	0	0	0
Negative control	54	Neg	Neg	0.00	0	0	0	0	0	0
Negative control	55	Neg	Neg	0.00	1	0	0	0	0	0
Turkey DPI 3	776	Neg	Neg	0.20	0	0	0	0	0	0
Turkey DPI 3	777	29.3	29.5	7.10	6	0	0	0	0	0
Turkey DPI 3	778	29.6	31.3	0.00	2	0	0	0	0	2
Turkey DPI 3	779	35.0	37.9	1.20	2	0	0	0	0	0
Turkey DPI 3	780	Neg	Neg	0.00	1	0	0	0	0	0
Turkey DPI 5	781	29.2	30.5	0.00	0	0	0	0	0	0
Turkey DPI 5	782	Neg	Neg	0.00	0	0	0	0	0	0
Turkey DPI 5	783	34.3	Neg	0.00	0	0	0	0	0	0
Turkey DPI 5	784	30.1	30.9	0.70	0	0	0	0	0	1
Turkey DPI 5	785	Neg	Neg	0.00	1	0	0	0	0	0
Bald eagle DPI 3	791	25.5	26.5	2.03	8	3	1	4	5	1
Bald eagle DPI 3	792	29.3	31.6	3.38	8	2	1	3	4	3
Bald eagle DPI 3	793	35.3	39.5	0.00	1	0	0	0	0	0
Bald eagle DPI 3	794	24.6	25.2	1.55	16	4	3	7	10	4
Bald eagle DPI 3	795	36.0	36.4	0.20	1	0	0	0	0	0
Bald eagle DPI 5	796	21.4	22.7	4.70	18	4	4	8	12	0
Bald eagle DPI 5	798	22.9	24.2	0.20	11	3	3	6	9	1
Bald eagle DPI 5	799	24.3	25.4	1.80	3	1	1	2	3	3
Bald eagle DPI 5	800	25.2	25.8	0.70	12	4	3	7	10	1
Bald eagle DPI 5	818	22.4	25.5	1.00	0	0	0	0	0	4
Raccoon DPI 3	56	34.7	31.9	0.40	0	0	0	0	1	0
Raccoon DPI 3	57	Neg	Neg	0.28	0	0	0	0	0	0
Raccoon DPI 3	58	18.6	18.2	3.70	16	4	3	7	2	1
Raccoon DPI 3	59	27.7	26.3	0.00	3	0	0	0	5	1
Raccoon DPI 3	60	28.2	27.9	0.50	11	2	1	3	5	1
Raccoon DPI 5	61	25.8	24.1	0.00	1	0	0	0	0	0
Raccoon DPI 5	62	28.1	26.3	0.00	1	0	0	0	0	0
Raccoon DPI 5	63	27.8	26.5	0.20	2	0	0	0	4	1
Raccoon DPI 5	64	29.6	28.3	0.00	7	0	0	0	2	2
Raccoon DPI 5	65	32.3	30.8	0.00	2	0	0	0	1	1
Redfox DPI 3	76	37.1	36.2	3.20	11	3	2	5	3	1
Redfox DPI 3	77	28.1	31.1	2.58	10	3	1	4	4	1
Redfox DPI 3	78	34.1	30.7	4.33	10	3	1	4	5	2
Redfox DPI 3	79	34.2	32.9	1.33	1	0	0	0	2	1
Redfox DPI 3	80	25.0	21.1	0.28	1	0	0	0	0	0
Redfox DPI 5	81	23.6	22.4	3.55	3	0	0	0	3	2

Group	Pig ID	BALF IAV PCR Ct	BALF H5 2.3.4.4 PCR Ct	Weighted Macroscopic Lung Lesion Score	Microscopic Pneumonia Score (0–20)	Conducting Airway Lung IHC Score (0–4)	Non-conducting Airway Lung IHC Score (0–4)	Cumulative Lung IHC Score (0–8)	Microscopic Tracheitis Score (0–8)	Trachea IHC Score (0–4)
Redfox DPI 5	82	30.7	28.1	0.00	2	0	0	0	3	1
Redfox DPI 5	83	27.7	26.3	1.43	3	1	1	2	3	1
Redfox DPI 5	84	Neg	Neg	0.28	1	0	0	0	3	0
Redfox DPI 5	85	28.8	27.7	1.78	7	2	2	4	1	0

*BALF, bronchoalveolar lavage fluid; Ct, cycle threshold; DPI, days postinoculation; IAV, influenza A viruses; IHC, immunohistochemistry.

Appendix 1 Table 2. qRT-PCR positive samples successfully sequenced by group and animal identification*

	BALF DPI		Nasal Swab DPI/DPC			
Group	3	5	1	3	5	7
Turkey		781, 784				
Bald eagle	791, 792, 793, 794	796, 798, 799, 800 818				
Raccoon	58, 59, 60	63, 65	61	61	61, 65	67, 69
Redfox	77, 78, 79, 80, 81	82, 83, 84		85, 86		

*BALF, bronchoalveolar lavage fluid; DPI, days postinoculation; DPC, days postcontact; qRT-PCR, quantitative reverse transcription PCR.

Appendix 1 Table 3. Influenza A virus mean RT-qPCR Ct values and mean viral titer of lung homogenate of a swine-adapted H1N1*

	Right Cranial Lung		Left Cranial Lung		Caudal Lung (Right or Left)		% Positive
DPI	PCR Ct Value	Viral Titer	PCR Ct Value	Viral Titer	PCR Ct Value	Viral Titer	Pigs
3	23.0 ± 1.0	6.5 ± 2.0	22.8 ± 1.1	6.3 ± 1.9	25.7 ± 1.2	5.0 ± 2.0	100
5	24.0 ± 1.4	5.3 ± 2.8	25.7 ± 1.7	5.3 ± 1.9	24.9 ± 1.3	5.6 ± 2.7	100

*Eight pigs per necropsy time point, infected with the same device and approximate dose of a swine-adapted influenza A virus strain (subtype H1N1, A/swine/Iowa/A02429950/2019, HA clade 1A.3.3.3) of a former study included for comparison (*10*). Ct, cycle threshold; DPI, days postinoculation.

Appendix 1 Table 4. Influenza A virus mean RT-qPCR Ct values and mean viral titer of nasal swab samples of a swine-adapted H1N1*

DPI	PCR Ct Value	Viral Titer	% Positive Pigs
1	30.4 ± 3.3	3.4 ± 1.4	100
3	28.5 ± 1.3	3.8 ± 0.9	100
5	28.8 ± 1.4	3.8 ± 1.0	100

*24 (day post-inoculation 1), 16 (day post-inoculation 3) and eight pigs (day post-inoculation 5), infected with the same device and approximate dose of a swine-adapted influenza A virus strain (subtype H1N1, A/swine/Iowa/A02429950/2019, HA clade 1A.3.3.3) of a former study included for comparison (*10*). Ct, cycle threshold; DPI, days postinoculation.

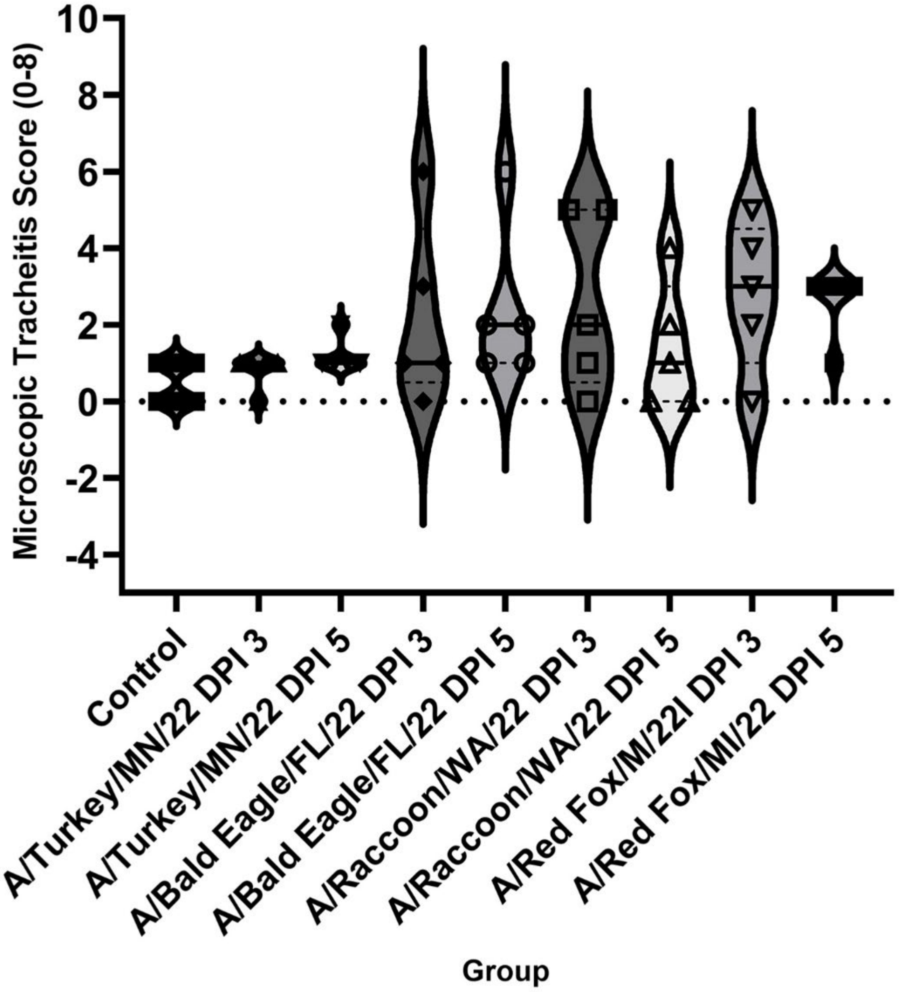

Appendix 1 Figure 1. Microscopic tracheitis score by strain and day post-inoculation (DPI). Median (solid line), quartiles (dashed line), and individual pig values (symbol) depicted. No statistically significant difference detected between strains by DPI.

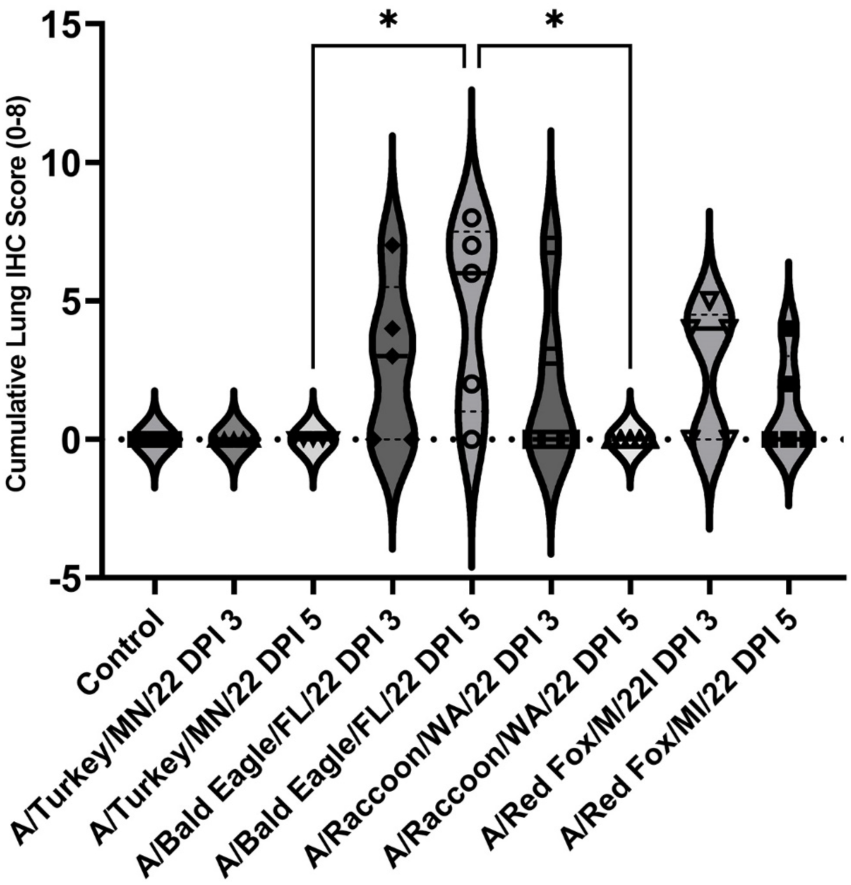

Appendix 1 Figure 2. Cumulative lung immunohistochemical score by highly pathogenic avian influenza strain and day post-inoculation (DPI). Median (solid line), quartiles (dashed line), and individual pig values (symbol) depicted. *$p < 0.05$.

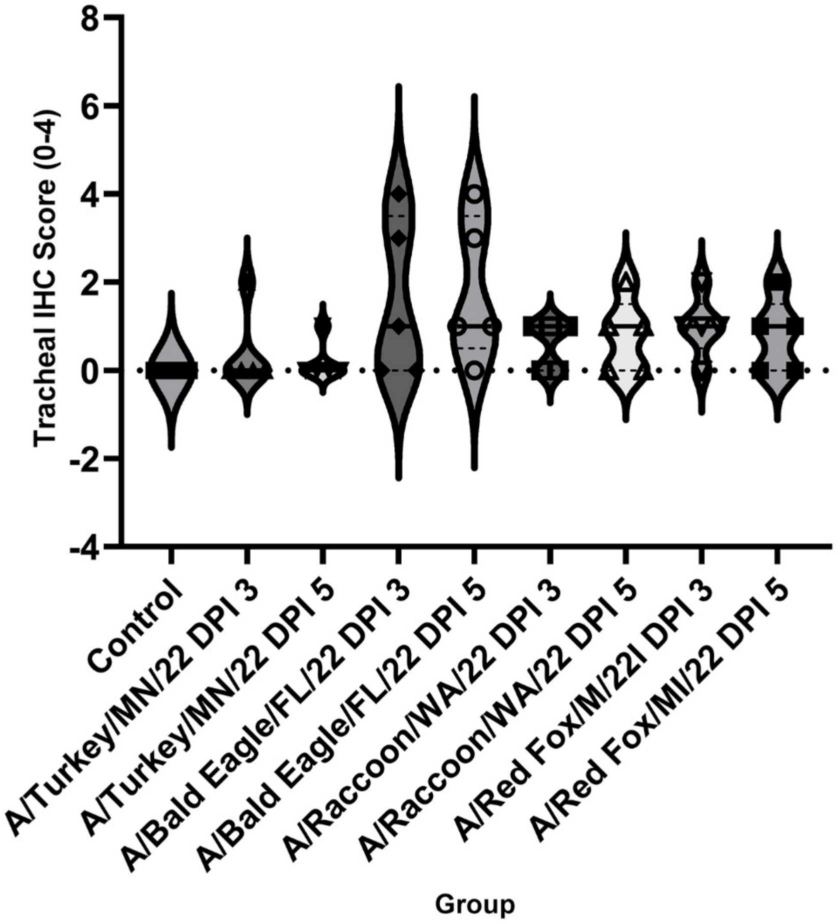

Appendix 1 Figure 3. Tracheal immunohistochemical score by highly pathogenic avian influenza strain and day post-inoculation (DPI). Median (solid line), quartiles (dashed line), and individual pig values (symbol) depicted. No statistically significant difference detected between strains by DPI.
